# Mental Confusion of Neurological Etiology in 41 cases

**DOI:** 10.1192/j.eurpsy.2022.2244

**Published:** 2022-09-01

**Authors:** S. Brahim, W. Bouali, S. Younes, M. Kacem, L. Zarrouk

**Affiliations:** 1 University Hospital of Mahdia, Psychiatry, chebba, Tunisia; 2 hospital Tahar sfar Mahdia, Epartment Of Psychiatry Mahdia, Mahdia, Tunisia; 3 University Hospital of Mahdia, Tunisia., Psychiatry, mahdia, Tunisia

**Keywords:** neurology, mental confusion

## Abstract

**Introduction:**

The confusional state is the clinical expression of a temporary acute cerebral decompensation. It is expressed by a global, fluctuating and reversible alteration of cognitive functions. It is a frequent reason for consultation in the emergency room.

**Objectives:**

To determine the epidemiology, neurological etiologies and their risk factors.

**Methods:**

Retrospective study based on the files of 41 hospitalized patients with confusional syndrome.

**Results:**

We collected 41 patients. The mean age was 72.9 years. The sex ratio was 1.25. The antecedents found were arterial hypertension and diabetes in 10 patients, a history of stroke was ischemic in 21 cases and hemorrhagic in 6 cases, cerebral metastasis in 5 cases, hepatic encephalopathy in 6 cases and a toxic cause in 4 cases. Mental confusion was acute in 23 patients and subacute in 18. The confusional manifestations observed were essentially temporospatial disorientation in 27 patients, obnubilation in 22 cases, difficulty in paying attention and concentrating in 24 cases, and vague and slow verbal expression in 11 cases. A metabolic disorder was found in 15 patients, such as hyperkalaemia (7 cases) and hyperglycaemia (8 cases). Etiological treatment was instituted with recourse to sedative treatment in 18 patients due to agitation. The evolution was marked by a total regression of confusion in 11 cases, lacunar amnesia in 7 cases, prolonged mental confusion in 6 cases and death in 3 patients.

**Conclusions:**

Elderly subjects are at risk due to the vulnerability of brain structures to pathologies and treatments associated with this period of life. Early treatment can improve the prognosis.

**Disclosure:**

No significant relationships.

